# Analysis of Changes in the Expression of Selected Genes from the *ABC* Family in Patients with Triple-Negative Breast Cancer

**DOI:** 10.3390/ijms24021257

**Published:** 2023-01-09

**Authors:** Anna Makuch-Kocka, Janusz Kocki, Anna Brzozowska, Jacek Bogucki, Przemysław Kołodziej, Anna Bogucka-Kocka

**Affiliations:** 1 Department of Pharmacology, Medical University of Lublin, 20-400 Lublin, Poland; 2 Department of Clinical Genetics, Medical University of Lublin, 20-400 Lublin, Poland; 3 Department of Radiotherapy, St. John of Dukla Lublin Region Cancer Center, 20-090 Lublin, Poland; 4 Department of Organic Chemistry, Medical University of Lublin, 20-093 Lublin, Poland; 5 Department of Biology and Genetics, Medical University of Lublin, 20-093 Lublin, Poland

**Keywords:** triple-negative breast cancer, multidrug resistance, *ABC* family genes, gene expression regulation

## Abstract

Triple-negative breast cancer (TNBC) is characterized by a lack of expression of hormone receptors (estrogen and progesterone), as cancer cells also do not overexpress the HER2 receptor. Due to their molecular profile, treatments for this type of breast cancer are limited. In some cases, the pharmacotherapy of patients with TNBC is hindered by the occurrence of multidrug resistance, which is largely conditioned by proteins encoded by genes from the *ABC* family. The aim of our study was to determine the expression profile of 14 selected genes from the *ABC* family using real-time PCR in 68 patients with TNBC by comparing the obtained results with clinical data and additionally using bioinformatics tools (Ualcan and The Breast Cancer Gene Expression Miner v4.8 (bc -GenExMiner v4.8)), as well as by comparing experimental data with data in the Cancer Genome Atlas (TCGA) database. Based on the conducted studies, we found different levels of gene expression depending on the age of patients, tumor sizes, metastases to lymph nodes, cell infiltration into adipose tissue, tumor stages, or lymphovascularinvasion. The results of the presented studies demonstrate the effect of the expression level of the studied genes on the clinical course and prognosis of patients with TNBC, and suggest how profiling the expression level of genes from the *ABC* family may be a useful tool in determining personalized TNBC treatment.

## 1. Introduction

Triple-negative breast cancer (TNBC) is characterized by a negative expression of the progesterone receptor (PR), the estrogen receptor (ER), and human epidermal growth factor receptor 2 (HER2) [1]. TNBC, as one of the molecular subtypes of breast cancer (BC), accounts for about 10–20% of all diagnosed cases [2,3,4,5]. TNBC is most often diagnosed in premenopausal women under 40 years of age [6]. TNBC is an aggressive type of BC; has a worse prognosis than other molecular subtypes of breast cancer; and is characterized by earlier recurrences and metastases, shorter patient survival, and high mortality [7,8,9,10]. Currently, the basic method of treating TNBC is chemotherapy; due to the lack of expression of ER, PR, and HER2 receptors, hormonal therapy and targeted molecular therapy do not show satisfactory results [11]. Unfortunately, many patients who receive chemotherapy develop drug resistance, which is a major obstacle to successful cancer treatment. Resistance to the applied cytostatics may account for up to 90% of cases of ineffective chemotherapy [12,13].

Multidrug resistance (MDR) is defined as the insensitivity of cancer cells to the applied chemotherapeutic drugs, which differ in their mechanism of action, structural structure, and target point. MDR is one of the main causes of chemotherapy failure and is responsible for increasing the mortality rate of cancer patients [14]. The mechanism underlying the emergence of the phenomenon of multidrug resistance is usually conditioned by many factors that are not fully understood [15,16].

There are two mechanisms of drug resistance in cancer cells: non-cellular and cellular. Non-cellular mechanisms result from the structure of solid tumors, the location of the tumor, the division rate of tumor cells, the degree of vascularization, the number of necrotic foci in the tissue, or the presence of natural barriers in the body. Cellular mechanisms are divided into classical and non-classical mechanisms. Classical mechanisms result from the physicochemical properties of the cell membrane, transmembrane transportation, and the chemical properties of cytostatics [17,18].

The proteins involved in the transportation of drugs in the human body and the induction of the MDR phenomenon in breast cancer are mainly proteins from the ABC family [13]. The most common cause of the MDR phenomenon is the active release of drugs from cells with the participation of ABC proteins, encoded by genes from the *ABC* (ATP binding cassette) family, e.g., P-glycoprotein [18]. Another mechanism that leads to the induction of multidrug resistance is the functional synergism of ABC proteins and p450 cytochromes. This is due to the increased affinity of CYP-produced metabolites (compared to the unmetabolized compound) for ABC proteins, e.g., cytochrome CYP3A4 and P-glycoprotein [18,19]. Non-classical mechanisms derive from biochemical pathways and drug metabolism. These mechanisms, regardless of the concentration of the drug reaching the cells, reduce the effectiveness of treatment. Non-classical mechanisms are revealed during the release, absorption, distribution, metabolism, and excretion of cytostatic agents in the body [18].

Many proteins encoded by genes from the *ABC* family are involved in the development of multidrug resistance in cancer, including breast cancer. In TNBC, proteins ABCC1, ABCC11, and ABCG2 have been identified as crucial in the development of MDR [20,21,22,23,24,25,26].

Taking into account the significant impact of MDR on the effectiveness of applied therapy in patients with TNBC and the proven role of ATP-binding cassette (ABC) transporters in the induction of resistance to chemotherapy, this paper presents the profiling of the expression level of selected genes from the *ABC* family in patients with TNBC. The paper describes changes in the expression level of selected 14 genes from the *ABC* family (*ABCA2*, *ABCA3*, *ABCB1*, *ABCB4*, *ABCB9*, *ABCC1*, *ABCC2*, *ABCC3*, *ABCC4*, *ABCC5*, *ABCC6*, *ABCC10*, *ABCC11*, and *ABCG2* genes) in patients diagnosed with TNBC. The obtained data were correlated with clinical data such as age; the invasion of fat tissue; lymphovascular invasion; tumor stage determined by the mitotic index, nuclear pleomorphism, and tubular differentiation (the Scarff–Bloom and Richardson (SBR) grading system) [27]; metastases to the lymph nodes; and tumor size. The results obtained from the experimental part were compared with data obtained from the Cancer Genome Atlas (TCGA) database using the bioinformatics tool The Breast Cancer Gene Expression Miner v4.8 (bc -GenExMiner v4.8) and Ualcan.

## 2. Results

### 2.1. The Expression Level of Selected Genes from the ABC Family in Patients with TNBC: Comparison of the Obtained Results with the Data Obtained from the TCGA Database

**Figure 1:**
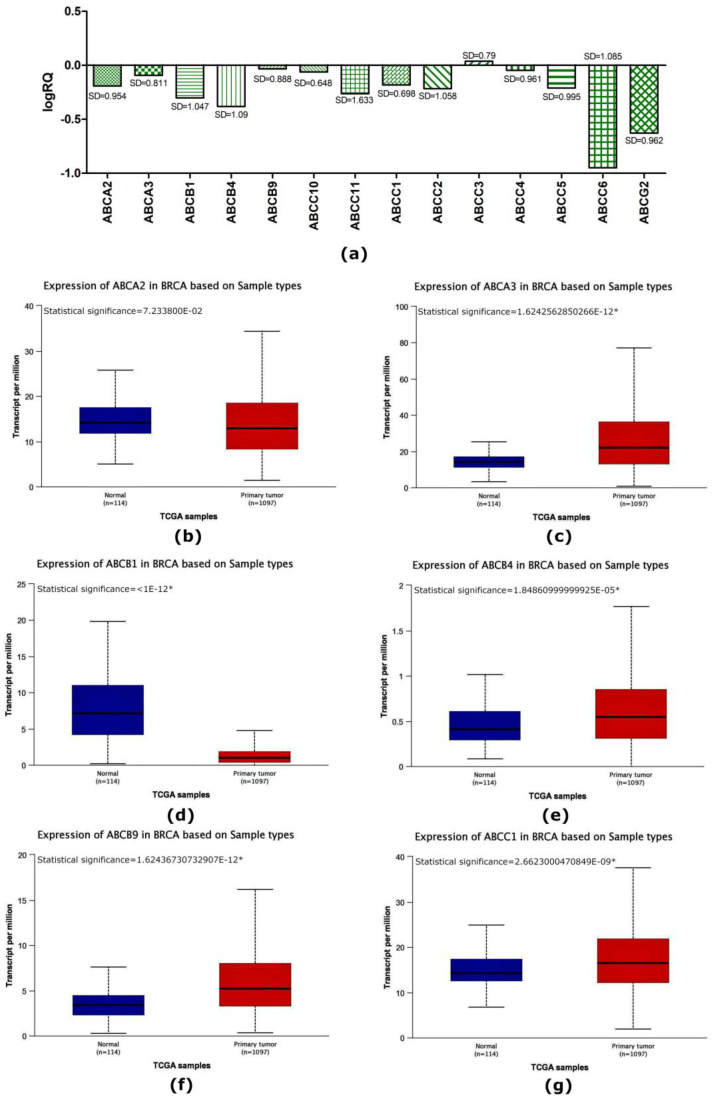
The average expression level of selected genes from the *ABC* family in patients with triple-negative breast cancer determined experimentally (**a**) and comparison of the expression level of the *ABCA2* (**b**), *ABCA3* (**c**), *ABCB1* (**d**), *ABCB4* (**e**), *ABCB9* (**f**), and *ABCC1* (**g**) genes in breast cancer patients compared to normal tissue obtained from the TCGA database using the Ualcan web tool. Statistically significant values are marked with *.

In patients with triple-negative breast cancer, the highest mean expression level compared to the normal tissue surrounding the tumor was noted for the *ABCC3* gene (M = 0.038216) (Figure 1a), while the lowest mean expression level was recorded for the *ABCC6* gene (M = −0.949418) (Figure 1a). For all marked genes from the *ABC* family, a reduced average level of expression was observed compared to the control, except for the *ABCC3* gene (M = 0.038216) (Figure 1a). Descriptive statistics for genes are presented in Table 1.

**Figure 2:**
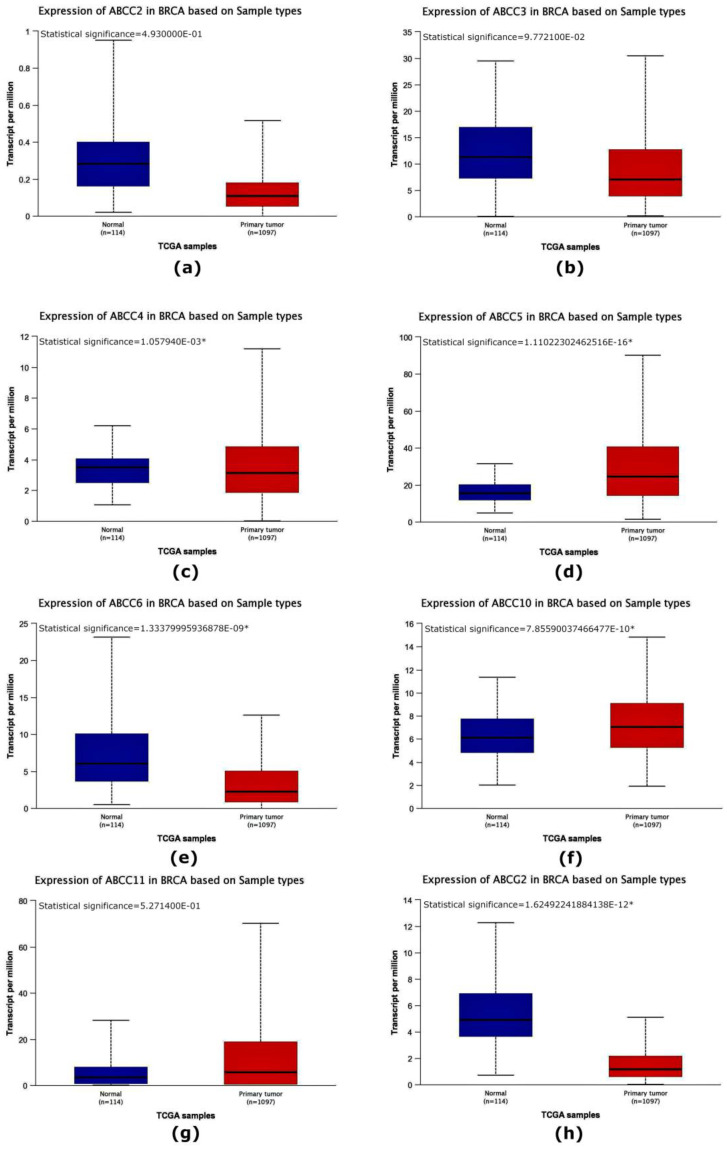
A comparison of the expression levels of the *ABCC2* (**a**), *ABCC3* (**b**), *ABCC4* (**c**), *ABCC5* (**d**), *ABCC6* (**e**), *ABCC10* (**f**), *ABCC11* (**g**), and *ABCG2* (**h**) genes in breast cancer patients compared to normal tissue obtained from the TCGA database using the Ualcan web tool. Statistically significant values are marked with *.

Data obtained from the TCGA database also showed a statistically significant lowered average expression level of the *ABCB1*, *ABCC4*, *ABCC6*, and *ABCG2* genes in patients with breast cancer compared to the control group (Figure 1 and Figure 2). In contrast to the average expression level of the *ABCC3* gene (Figure 2) obtained during the experiments, the data obtained from the TCGA database showed a decrease in the average expression level of this gene, but these values were not statistically significant compared to the control group. The average expression levels of the *ABCA3*, *ABCB4*, *ABCB9*, *ABCC1*, *ABCC5*, and *ABCC10* genes (Figure 1 and Figure 2) obtained from the TCGA database were elevated compared to normal tissue, which was not observed in the results of the experimental part. Inconsistencies in the data obtained experimentally in comparison with the data deposited in the TCGA may result from differences in the expression levels of estrogen, progesterone, and HER2 receptors in cancers in patients from the study group.

### 2.2. The Relationship between the Selected Genes from the ABC Family and a Comparison of the Results Obtained from the Experimental Part with the Bioinformatics Analysis Data Results from the TCGA and SCAN-B Database

**Figure 3:**
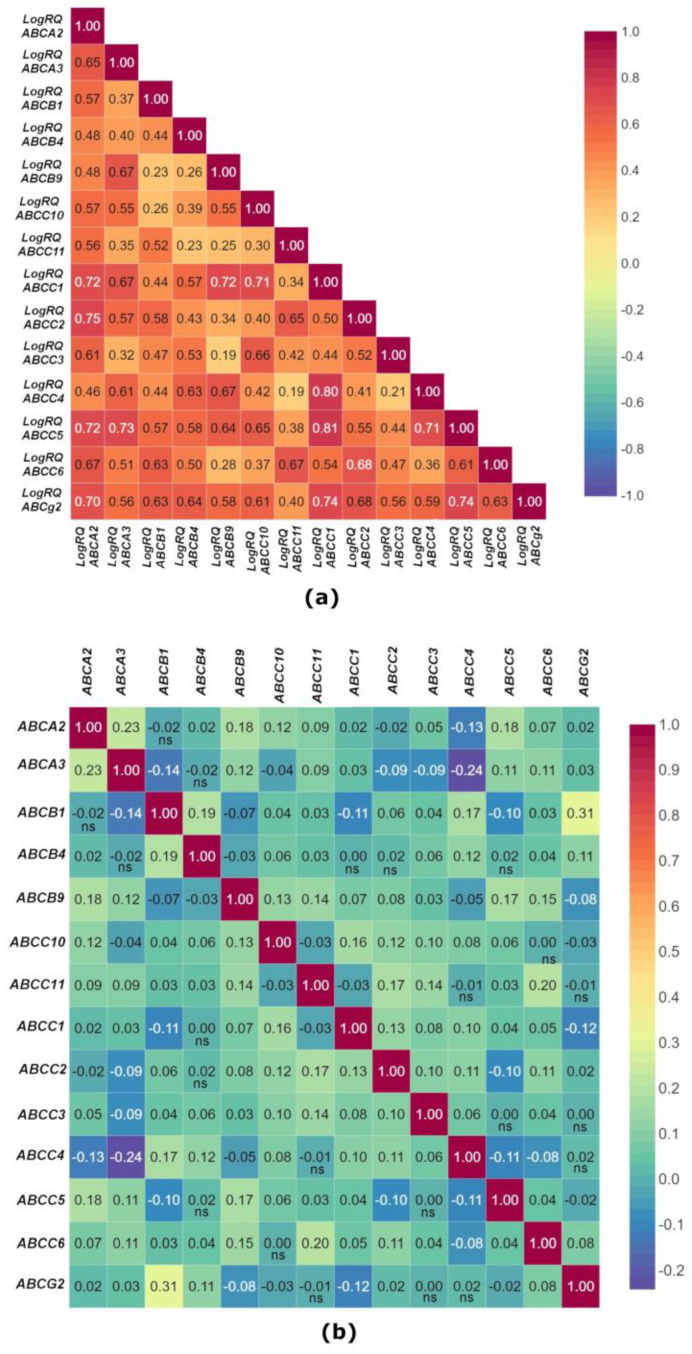
The figure shows correlation analyses of mean expression values of selected genes from the *ABC* family in patients with triple-negative breast cancer (**a**) and correlation analyses of mean expression values of tested genes in breast cancer obtained from the TCGA and SCAN-B database using Breast Cancer Gene Expression Miner v4.8 online tool (r—Pearson’s correlation coefficient) (**b**). ns: No significant difference.

A correlation analysis of the mean expression values of selected genes from the ABC family showed that there is a statistically significant positive correlation between all the tested genes. The strongest positive correlations occur between the genes *ABCC1* and *ABCA2* (r = 0.72), *ABCC2* and *ABCA2* (r = 0.75), *ABCC5* and *ABCA2* (r = 0.72), *ABCG2* and *ABCA2* (r = 0.70), *ABCC5* and *ABCA3* (r = 0.73), *ABCC1* and *ABCB9* (r = 0.72), *ABCC1* and *ABCC10* (r = 0.71), *ABCC4* and *ABCC1* (r = 0.80), *ABCC5* and *ABCC1* (r = 0.81), *ABCG2* and *ABCC1* (r = 0.74), *ABCC5* and *ABCC4* (r = 0.71), and *ABCG2* and *ABCC5* (r = 0.74). The lowest values of the correlation coefficient (r = 0.19) were found for the *ABCC3* and *ABCB9* genes and the *ABCC4* and *ABCC11* genes (Figure 3a).

The data analysis obtained from the TCGA and SCAN-B confirmed statistically significant positive correlations between the experimentally determined genes, with the exception of the following gene pairs: *ABCA2* and *ABCB1*, *ABCC2* and *ABCA2*, *ABCC4* and *ABCA2*, *ABCB1* and *ABCA3*, *ABCB4* and *ABCA3*, *ABCC10* and *ABCA3*, *ABCC2* and *ABCA3*, *ABCC3* and *ABCA3*, *ABCC4* and *ABCA3*, *ABCB9* and *ABCB1*, *ABCC1* and *ABCB1*, *ABCC5* and *ABCB1*, *ABCB9* and *ABCB4*, *ABCC4* and *ABCB9*, *ABCG2* and *ABCB9*, *ABCC11* and *ABCC10*, *ABCG2* and *ABCC10*, *ABCC1* and *ABCC11*, *ABCG2* and *ABCC1*, *ABCC5* and *ABCC2*, *ABCC5* and *ABCC4*, *ABCC5* and *ABCC4*, and *ABCG2* and *ABCC5* (Figure 3b).

### 2.3. Analysis of the Relationship between the Expression of Selected Genes from the ABC Family and Clinical Data (Age, Invasion of Fat Tissue, Lymphovascular Invasion, SBR Grade, Metastases to the Lymph Nodes, and Tumor Size)

#### 2.3.1. Age

**Figure 4:**
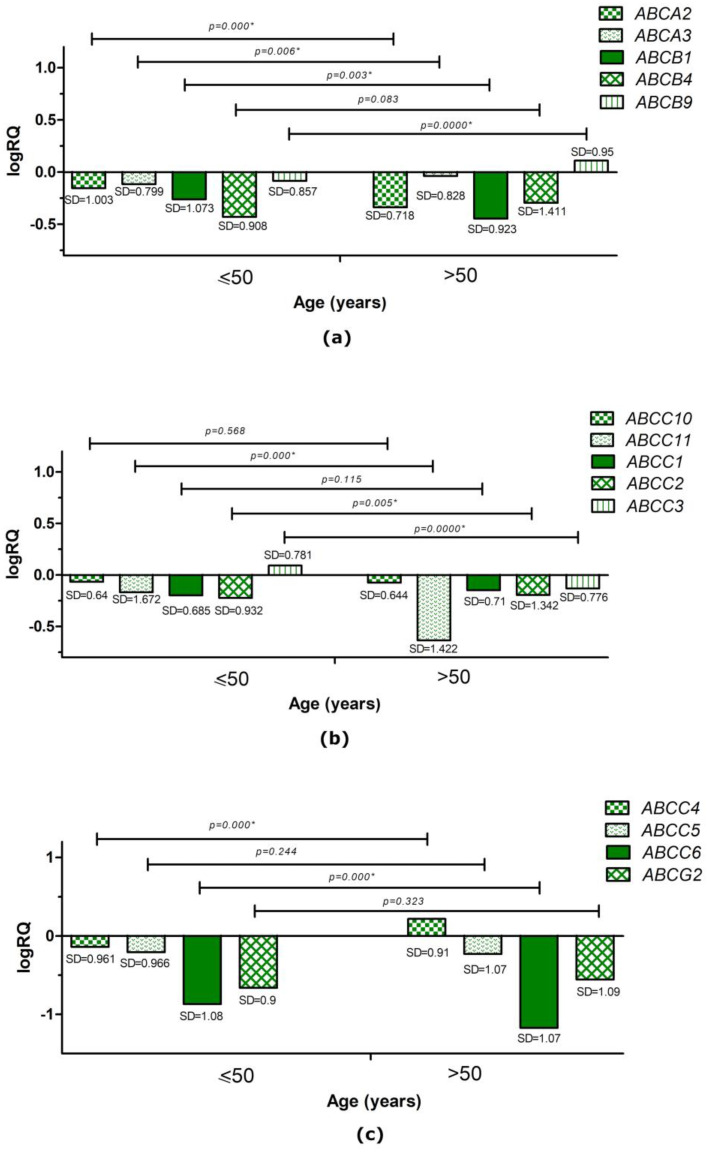
Mean expression level (logRQ) of the *ABCA2*, *ABCA3*, *ABCB1*, *ABCB4*, and *ABCB9* genes (**a**); the *ABCC10*, *ABCC11*, *ABCC1*, *ABCC2*, and *ABCC3* genes (**b**); and the *ABCC4*, *ABCC5*, *ABCC6*, and *ABCG2* genes (**c**) in triple-negative breast cancer tissue compared to tissue normal in age groups (≤50 years, >50 years). * Significance level of the U Mann–Whitney test.

The analysis performed with the U Mann–Whitney test showed that the average expression level of the *ABCA2* (*p* = 0.000), *ABCB1* (*p* = 0.003), *ABCC11* (*p* = 0.000), *ABCC3* (*p* = 0.000), and *ABCC6* (*p* = 0.000) genes was significantly statistically higher in patients aged 50 years or younger, while the mean expression level of the *ABCA3* (*p* = 0.006), *ABCB9* (*p* = 0.000), *ABCC2* (*p* = 0.005), and *ABCC4* (*p* = 0.000) genes was statistically significantly higher in patients over 50 years of age compared to younger patients (Figure 4).

Data obtained from the TCGA and the SCAN-B database showed a statistically significantly higher average expression level of the *ABCG2* (*p* = 0.0465), *ABCC11* (*p* < 0.0001), *ABCC6* (*p* < 0.001), *ABCC5* (*p* = 0.0012), and *ABCB9* (*p* = 0.0152) genes in patients over 51 years of age than in younger patients, while for the *ABCC10* (*p* < 0.0001), *ABCC4* (*p* < 0.0001), *ABCC1* (*p* = 0.0087), and *ABCB1* (*p* = 0.0014) genes, the mean expression level was higher in patients 51 years of age or younger (Figures S1–S3). Data on the *ABCB9* and *ABCB1* genes confirmed our results, while for the *ABCC4*, *ABCC6*, and *ABCC11* genes, the average expression levels depending on the age group generated from TCGA and the SCAN-B database differed from the experimental data. Differences in the obtained results may be due to the different sizes of the study groups or different age selection criteria when separating the groups for analysis.

#### 2.3.2. Invasion of the Fat Tissue

**Figure 5:**
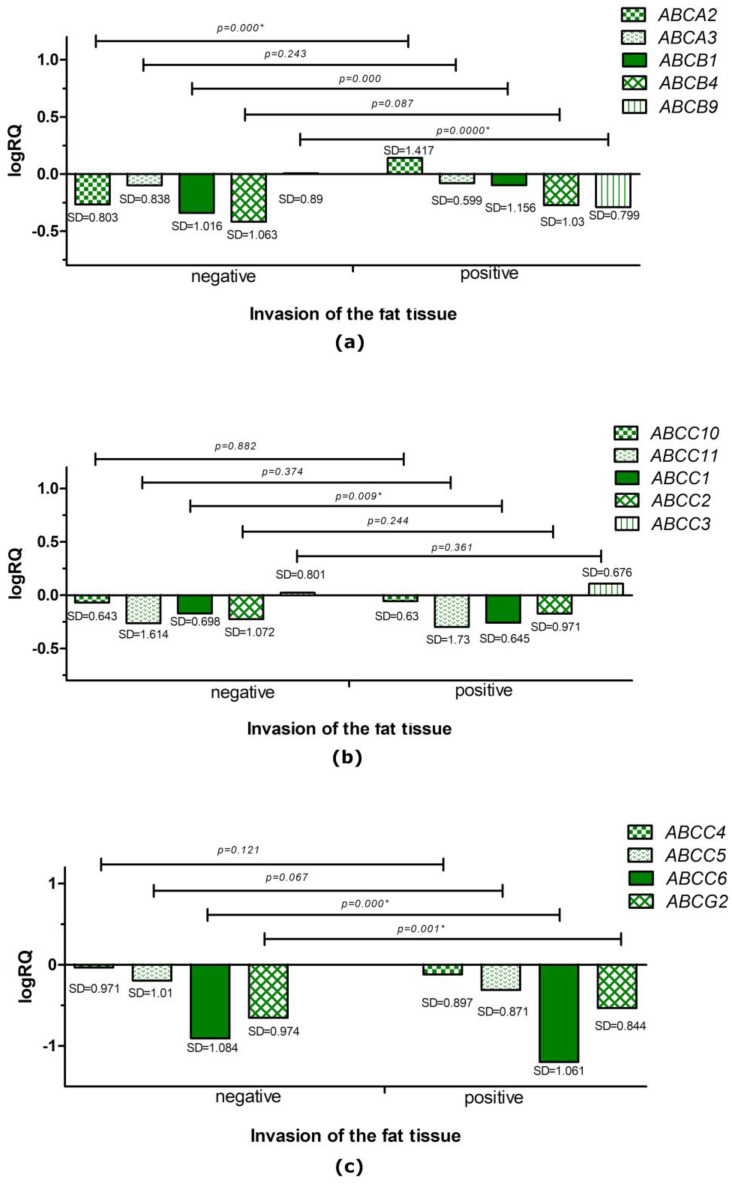
Mean expression level (logRQ) of the *ABCA2*, *ABCA3*, *ABCB1*, *ABCB4*, and *ABCB9* genes (**a**); the *ABCC10*, *ABCC11*, *ABCC1*, *ABCC2*, and *ABCC3* genes (**b**); and the *ABCC4*, *ABCC5*, *ABCC6*, and *ABCG2* genes (**c**) in triple-negative breast cancer tissue compared to tissue normal in groups based on the presence or absence of cancer cells in fat tissue. * Significance level of the U Mann–Whitney test.

Statistical analysis performed with the U Mann–Whitney test showed a statistically significantly higher expression level of the *ABCB9* (*p* = 0.000), *ABCC1* (*p* = 0.009), and *ABCC6* (*p* = 0.000) genes in patients without tumor cell invasion into fat tissue. The average expression level of the *ABCA2* (*p* = 0.000), *ABCB1* (*p* = 0.000), and *ABCG2* (*p* = 0.001) genes was statistically significantly higher in patients with diagnosed tumor cell infiltration into fat tissue compared to patients without tumor cells in the fat tissue (Figure 5).

In the TCGA database, no information was found on the expression level of the examined genes depending on the presence or absence of tumor cell infiltration in fat tissue.

#### 2.3.3. Lymphovascular Invasion

**Figure 6:**
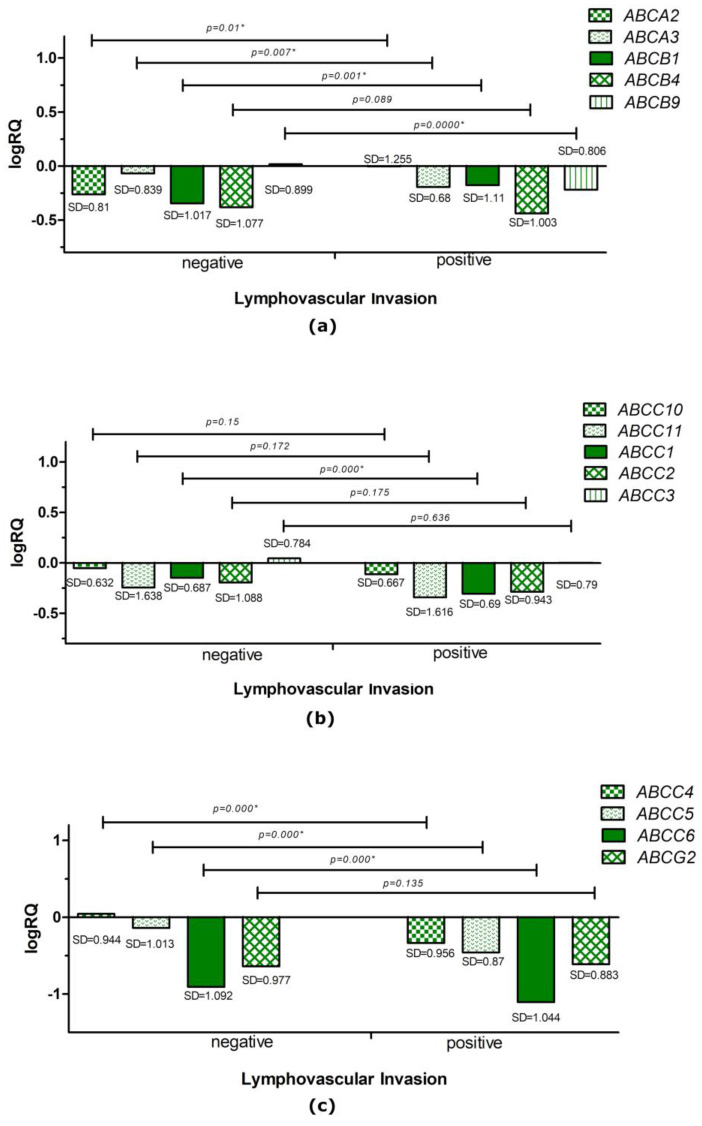
Mean expression level (logRQ) of the *ABCA2*, *ABCA3*, *ABCB1*, *ABCB4*, and *ABCB9* genes (**a**); the *ABCC10*, *ABCC11*, *ABCC1*, *ABCC2*, and *ABCC3* genes (**b**); and the *ABCC4*, *ABCC5*, *ABCC6*, and *ABCG2* genes (**c**) in triple-negative breast cancer tissue compared to tissue normal in groups based on the presence or absence of lymphovascular invasion. Statistically significant values are marked with *.

The analysis performed with the U Mann–Whitney test showed that the average expression level of the *ABCA2* (*p* = 0.01) and *ABCB1* (*p* = 0.001) genes was statistically significantly higher in patients associated with the lymphatic vessel infiltration of cancer cells. The average expression level of the *ABCA3* (*p* = 0.007), *ABCB9* (*p* = 0.000), *ABCC1* (*p* = 0.000), *ABCC4* (*p* = 0.000), *ABCC5* (*p* = 0.000), and *ABCC6* (*p* = 0.000) genes was statistically significantly higher in patients without tumor cell infiltration in the lymph vessels (Figure 6).

The statistical analysis of data from the TCGA and the SCAN-B database showed a statistically significant increase in the expression of the *ABCB9* (*p* < 0.0001), *ABCC2* (*p* = 0.0114), *ABCC3* (*p* < 0.0001), *ABCC5* (*p* < 0.0001), *ABCC10* (*p* = 0.0269), and *ABCC11* (*p* < 0.0001) genes in patients with lymphovascular invasion (nodal status). The data obtained experimentally for the *ABCB9* and *ABCC3* genes differ from the data generated from the TCGA and the SCAN-B database (Figures S4–S6).

#### 2.3.4. The Scarff–Bloom and Richardson (SBR) Grading System

**Figure 7:**
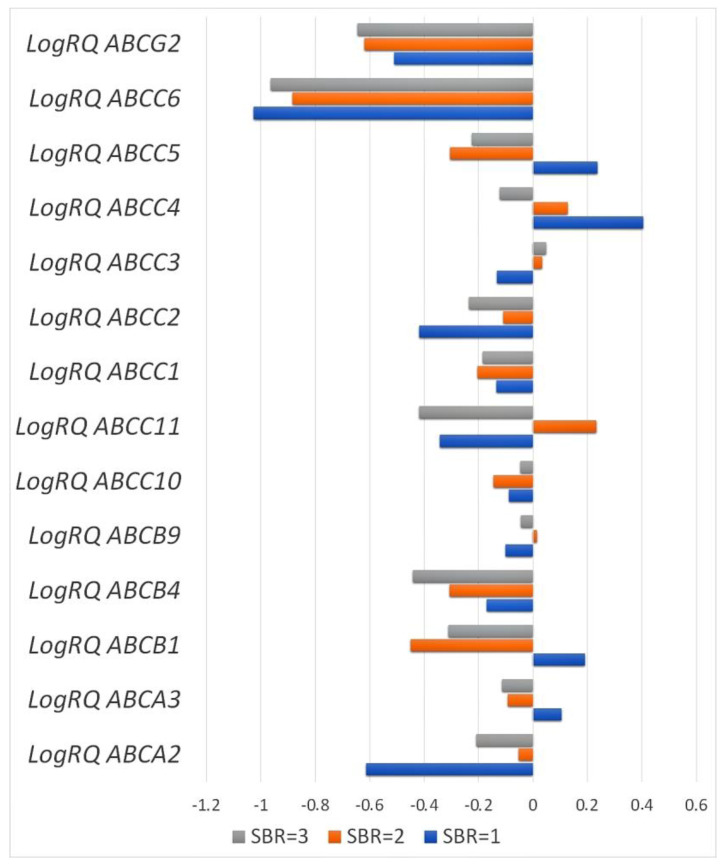
Mean expression values of selected genes from the *ABC* family in patients classified into the SBR1, SBR2, and SBR3 groups according to tumor stage (Scarff–Bloom and Richardson (SBR) scale).

The H Kruskal–Wallistest, together with the analysis of multiple comparisons, showed statistically significant differences in the expression of the *ABCA2*, *ABCA3*, *ABCB1*, and *ABCC5* genes in patients from the SBR1 and SBR2 groups and the SBR1 and SBR3 groups, while there was no statistically significant difference in the patients classified into the SBR2 and SBR3 groups. Statistically significant differences in the expression of the *ABCB4* gene were noted in patients from the SBR1 and SBR2 groups and the SBR2 and SBR3 groups. In the case of the average value of *ABCC10* gene expression, statistically significant differences were observed among patients from the SBR2 and SBR3 groups. For the mean value of *ABCC4* gene expression, statistically significant differences were observed in patients from the SBR1 and SBR2 groups, the SBR1 and SBR3 groups, and the SBR2 and SBR3 groups. Statistically significant differences in the mean expression of *ABCC11* and *ABCC2* genes were observed in patients from the SBR1 and SBR2 groups and the SBR2 and SBR3 groups (Table 2, Figure 7).

#### 2.3.5. Metastases to the Lymph Nodes

**Figure 8:**
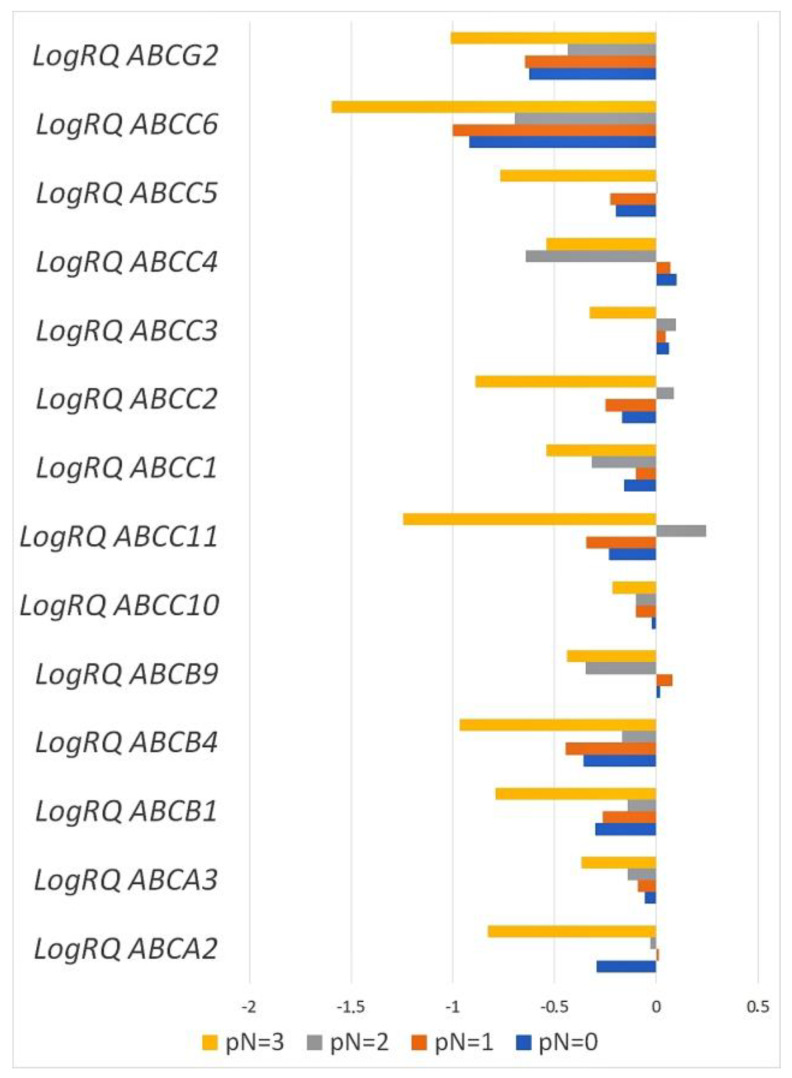
Mean expression values of selected genes from the *ABC* family in patients classified into 4 groups depending on the presence of metastases to lymph nodes (pN0—no metastases to the regional lymph nodes, pN1—micrometastases or metastases in 1–3 axillary lymph nodes, pN2—metastases in 4–9 axillary lymph nodes, and pN3—metastases in 10 or more axillary lymph nodes).

Statistical analysis showed statistically significant differences in the expression level the *ABCA3*, *ABCB9*, and *ABCC4* genes in patients classified into the pN0 and pN2 groups, the pN0 and pN3 groups, the pN1 and pN2 groups, and the pN1 and pN3 groups. Statistically significant differences were also observed in patients classified into the pN0 and pN2 groups, the pN0 and pN3 groups, the pN1 and pN2 groups, the pN1 and pN3 groups, and the pN2 and pN3 groups in terms of the expression level of the *ABCC11*, *ABCC1*, *ABCC2*, and *ABCC6* genes. Statistically significant differences in the expression levels of the *ABCB1*, *ABCC3*, and *ABCC5* genes were found in patients from the pN0 and pN3 groups, the pN1 and pN3 groups, and the pN2 and pN3 groups. The H Kruskal–Wallis test with multiple comparisons also showed statistically significant differences in the level of *ABCB4* gene expression in patients from the pN0 and pN1 groups, the pN0 and pN2 groups, the pN0 and pN3 groups, the pN1 and pN3 groups, and the pN2 and pN3 groups. For the *ABCC10* gene, a significant statistical difference in the level of expression occurred in patients from the pN0 and pN1 groups and the pN0 and pN3 groups. The ABCA2 gene expression values differed statistically significantly in patients in the pN0 and pN1 groups, the pN0 and pN2 groups, the pN1 and pN2 groups, the pN1 and pN3 groups, and the pN2 and pN3 groups. For the *ABCG2* gene, statistical significance was demonstrated in the pN0 and pN2 groups, the pN0 and pN3 groups, the pN1 and pN3 groups, and the pN2 and pN3 groups (Figure 8, Table S1).

Statistical analysis of data obtained from TCGA confirmed statistically significant differences in the expression level of the *ABCB1* and *ABCC3* genes in patients classified into pN0 and pN3 groups, the *ABCA2* gene in patients from the pN0 and pN1 groups, the *ABCB9* gene in patients from the pN0 and pN3 groups, the *ABCC4* gene in patients from the pN0 and pN3 groups and the pN1 and pN3 groups, and the *ABCC6* gene in patients from the pN0 and pN3 groups and the pN1 and pN3 groups. In addition, the analysis using the Ualcan online tool showed statistically significant differences in the expression level of the *ABCB9* gene in patients from the pN0 and pN1 groups and the pN1 and pN2 groups; the *ABCC3* gene in patients from the pN0 and pN1 groups and the pN1 and pN2 groups; and the *ABCC5* gene in patients from the pN0 and pN1 groups, the pN0 and pN2 groups, and the pN1 and pN2 groups, which was not shown in the experimental part (Table S2).

#### 2.3.6. Primary Tumor Size

**Figure 9:**
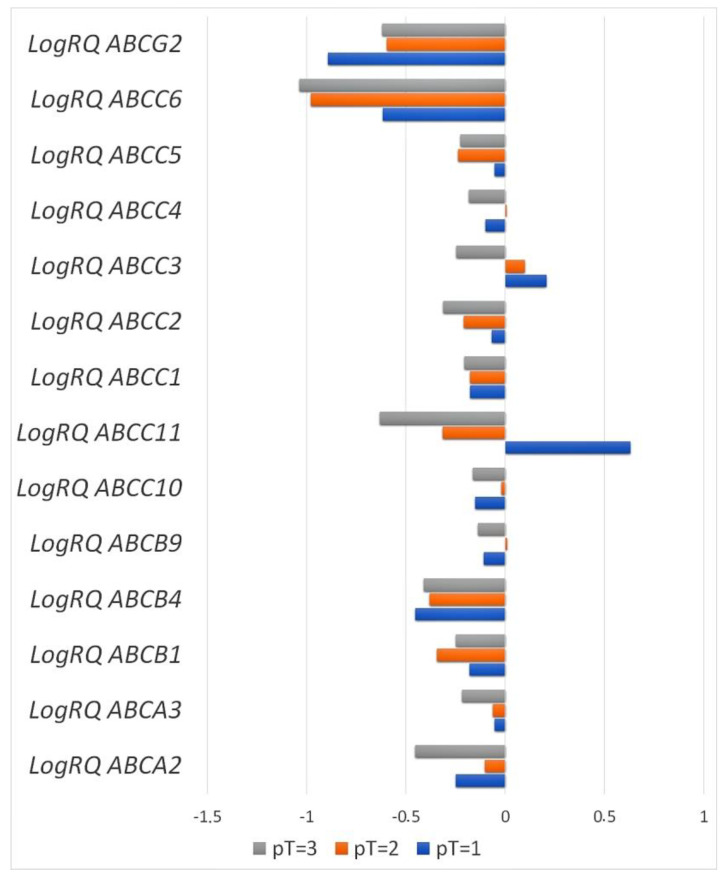
The average expression level of the tested genes from the *ABC* family in patients classified into the T1, T2, or T3 groups, depending on the size of the primary tumor (T1—patients with a primary tumor size ≤ 20 mm, T2—patients with a primary tumor size > 20 mm but ≤50 mm, and T3—patients with a primary tumor size greater than 50 mm).

The analysis showed statistically significant differences in the mean expression of the *ABCA2*, *ABCA3*, *ABCB4*, and *ABCC3* genes in patients classified into the T1 and T3 groups and the T2 and T3 groups. Differences in the mean expression values of the *ABCB1*, *ABCC2*, *ABCC5*, *ABCC6*, and *ABCG2* genes were statistically significant for patients from the T1 and T2 groups and the T1 and T3 groups. In the case of the *ABCB9* and *ABCC4* genes, there was a statistically significant difference in the level of expression in patients belonging to the T2 and T3 groups. The difference in the mean expression level of the *ABCC10* gene was statistically significant in the case of patients from the T1 and T2 groups and the T2 and T3 groups, while a significant difference in expression for the *ABCC11* gene was demonstrated between patients from all groups, separated on the basis of tumor size (Figure 9, Table 3).

The Cancer Genome Atlas database does not contain information on the expression level of the tested genes from the *ABC* family in classified patients according to the size of the primary tumor.

### 2.4. Analysis of the Impact of the Expression Level of Selected Genes from the ABC Family on the Overall Survival of Patients with Triple-Negative Breast Cancer—Analysis of Data from the TCGA

**Figure 10:**
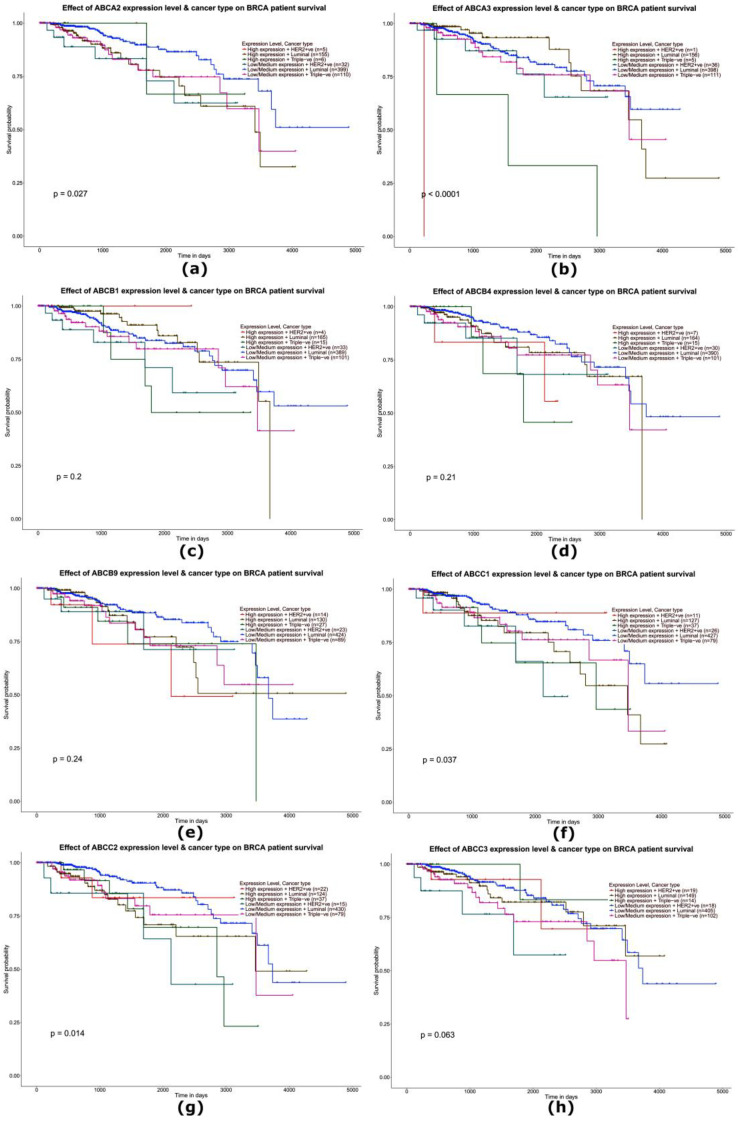
Survival curves (Kaplan–Meier plot) including ab assessment of the prognostic significance of the *ABCA2* (**a**), *ABCA3* (**b**), *ABCB1* (**c**), *ABCB4* (**d**), *ABCB9* (**e**), *ABCC1* (**f**), *ABCC2* (**g**), and *ABCC3* (**h**) genes in patients with breast cancer (different molecular subtypes). The data were generated using the online tool Ualcan.

**Figure 11:**
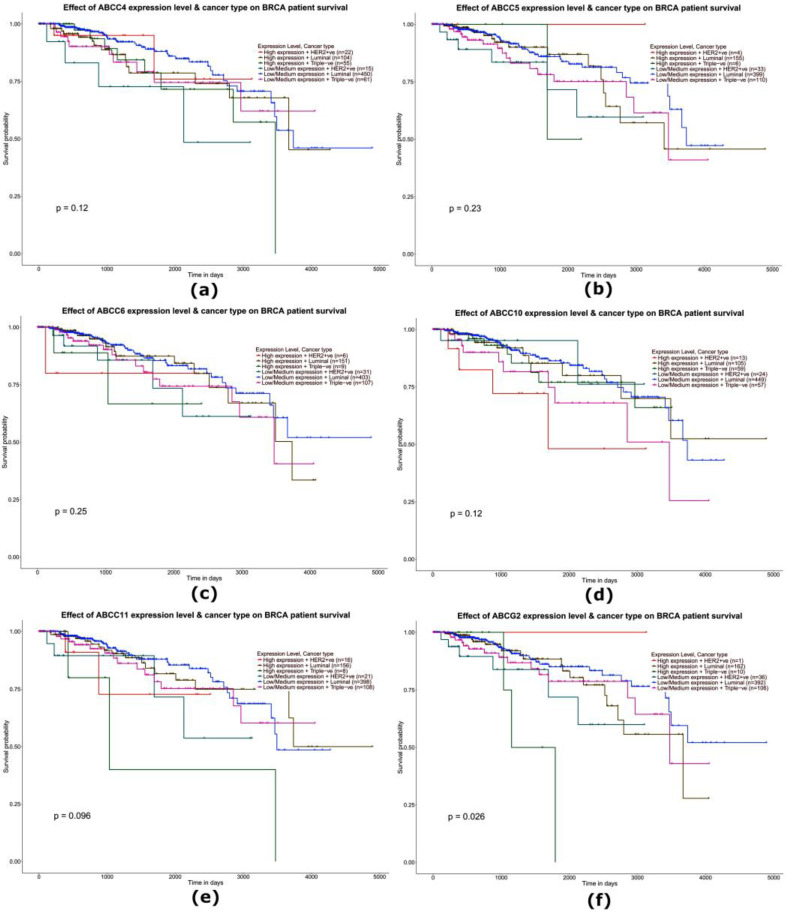
Survival curves (Kaplan–Meier plotter) including an assessment of the prognostic significance of the *ABCC4* (**a**), *ABCC5* (**b**), *ABCC6* (**c**), *ABCC10* (**d**), *ABCC11* (**e**), and *ABCG2* (**f**) genes in patients with breast cancer (different molecular subtypes). The data were generated using the online tool Ualcan.

The influence of the expression level of the studied genes on the overall survival (OD) of TNBC patients was studied using a Kaplan–Meier plotter. The analysis showed a statistically significant effect of the increase in the expression level of the *ABCA2* (*p* = 0.027), *ABCA3* (*p* < 0.0001), *ABCC1* (*p* = 0.037), *ABCC2* (*p* = 0.014), ABCC3 (*p* = 0.063), and *ABCG2* (*p*= 0.026) genes for shorter patient survival. The expression level of the remaining *ABC* family genes tested did not show a statistically significant effect on patient survival (*p* > 0.05) (Figure 10 and Figure 11).

## 3. Discussion

Breast cancer is characterized by high heterogeneity in terms of the clinical course, prognosis, method of treatment, and histopathological features, which is why it is so important to determine the subtype of the cancer before choosing a treatment [28]. The selection of the appropriate therapy in breast cancer is based on a combination of the cancer staging system, the patient’s age at diagnosis, the tumor histotype and stage, the hormone receptor status, and the TNM classification. The implementation of effective treatment with a low level of toxicity in patients requires the development of selective and individualized therapies based on the molecular and clinical features of the tumor. The development of such therapies should be based on the knowledge of the benefits and potential toxic effects of each therapeutic regimen [29].

One of the greatest challenges of 21st century medicine is the selection of appropriate pharmacotherapy that would allow for the remission of the disease. One of the reasons breast cancer chemotherapy fails is the occurrence of the phenomenon of multidrug resistance associated with proteins encoded by genes from the *ABC* family [30].

In our study, we showed that the expression level of the *ABCA2*, *ABCA3*, *ABCB1*, *ABCB4*, *ABCB9*, *ABCC1*, *ABCC2*, *ABCC4*, *ABCC5*, *ABCC6*, *ABCC10*, *ABCC11*, and *ABCG2* genes was reduced in relation to the normal tissue surrounding the tumor (control) and only the average level of expression *ABCC3* gene was elevated compared to controls (Figure 1 and Figure 2). Analyzing clinical data, we showed that an expression of the *ABCA3*, *ABCB9*, *ABCC2*, and *ABCC4* genes was statistically significantly higher in patients over 50 years of age (Figure 4). A higher expression level of the *ABCA2*, *ABCB1*, and *ABCG2* genes was found in patients with a confirmed invasion of neoplastic cells into fat tissue (Figure 5), and the expression of the *ABCA2* and *ABCB1* genes was higher in patients with lymphovascula invasion (Figure 6). Statistically significant differences in the expression of the examined genes were also observed in patients classified into groups depending on the size of the primary tumor, metastases to lymph nodes, or the SBR scale (Figure 7, Figure 8 and Figure 9).

The expression level of the *ABCB1* gene that we determined confirms the results obtained by João Marcos de Azevedo Delou and colleagues. Researchers characterized changes in the expression level of the *ABCB1* gene in a breast tumor compared to benign breast tissue surrounding the tumor. In total, 712 women were qualified for the study, 62 of whom were diagnosed with triple-negative breast cancer. The analysis of the results showed that a decrease in the expression of the *ABCB1* gene in patients diagnosed with triple-negative breast cancer is associated with the early stage of the disease and shorter survival of patients. The authors do not provide a possible causal mechanism explaining the effect of a decrease in the level of *ABCB1* gene expression on the proliferation, migration, survival, or invasion of cancer cells. It is believed that a decrease in the *ABCB1* gene expression in breast cancer cells may be a consequence of genomic instability in cancer cells, especially in triple-negative breast cancer [31].

In 2013, Hlaváč and colleagues published the results of their research on the search for new biomarkers of prognosis and/or predicting drug resistance among genes belonging to the ABC family in breast cancer patients (without division into molecular subtypes). For this purpose, the expression profile of 49 genes belonging to the *ABC* family was analyzed in the neoplastic tissue of breast cancer and in the normal tissue surrounding the tumor, treated at the Clinic Oncology Surgery in Prague. The obtained results indicated a significant increase in the expression level of the *ABCA2*, *ABCA3*, *ABCB9*, *ABCC4*, *ABCC1*, *ABCC5*, *ABCC11*, and *ABCC10* genes in tumors compared to control tissues. For the *ABCB1* and *ABCC6* genes, the expression level was significantly reduced in the tumors. There were no significant changes in expression between the tumor and control tissues in the ABCB4, ABCA13, ABCC2, ABCC3, and ABCG2 genes. The expression levels of the ABCC10 and ABCC1 genes were higher in ER-negative patients than in ER-(+) patients. The expression level of the ABCC11 gene was higher in tumors with ER receptor expression. The expression levels of the ABCC11 and ABCA2 genes were significantly higher in patients with an expression of the progesterone receptor [32]. Differences in the gene expression levels in the work described above and in the assays performed by us may result from the fact that the level of expression was determined in a specific molecular subtype of BC–TNBC in our studies; however, in the described work, there is no separation of TNBC samples.

Balaji and colleagues examined the expression level of the *ABCC1* and *ABCC3* genes in breast cancer patients (without division into molecular subtypes) and assessed their role in inducing the phenomenon of resistance to anticancer drugs. They observed that the expression level of the *ABCC1* and *ABCC3* genes is elevated in breast cancer tissue, especially after treatment with anticancer drugs. The overexpression of the *ABCC1* and *ABCC3* genes led to a decrease in drug retention. The study showed that, like the *ABCC1* gene, the *ABCC3* gene was overexpressed in stage III primary breast cancer. Importantly, in in vivo and in vitro studies, drug treatment led to a further increase in *ABCC1* and *ABCC3* gene expression levels. In our studies, we also showed an increased expression level of the *ABCC3* gene in the tumor tissue of patients with TNBC compared to controls, which may indicate the gene’s role in inducing multidrug resistance in breast cancer cells. [33].

In conclusion, based on the experiments carried out, it can be concluded that the expression level of selected genes from the *ABC* family is associated with the molecular subtype of breast cancer as well as clinical features. Gene expression profiling is an important tool for assessing the genetic diversity of breast cancer and may influence the selection of appropriate effective therapy for women with triple-negative breast cancer. The high costs of the expression level profiling of all genes from the *ABC* family in patients with TNBC prevent the use of this method in routine hospital practice. However, based on literature data on the determination of the expression level of selected proteins encoded by genes from the *ABC* family in patients with TNBC and the results presented by us, it can be assumed that good prognostic markers of the course of TNBC and response to treatment in oncological patients can determine the expression level of the *ABCC1*, *ABCC11*, and *ABCG2* genes [20,21,22,23,24,25,26]. More large-scale prospective studies as well as in vitro and in vivo functional studies are needed to assess the potential of *ABC* family genes as markers of the clinical course of TNBC and treatment responses in cancer patients.

## 4. Materials and Methods

### 4.1. Characteristics of Patients Qualified for the Study

Sixty-eight patients diagnosed with triple-negative breast cancer treated at the Oncology Center in Lublin were qualified for the study. The patients participating in the study gave their informed consent, and the study was approved by the Ethics Committee of the Medical University of Lublin (decision number: KE-0254/216/2014). The research was carried out in accordance with the Helsinki Declaration. The patients included in the study had no comorbidities, did not receive neoadjuvant chemotherapy, and did not indicate the presence of neoplasms in their family members in the history. Detailed clinical data of the patients are presented in Table 4. To determine the histological type of breast cancer, pathological lymph node metastases (pTNM), and tumor advancement, the 4th edition Classification of Tumors by the World Health Organization (WHO) for breast tumors [34], the 7th edition of the TNM classification of the American Joint Committee on Cancer (AJCC) [35], and the Scarff–Bloom and Richardson (SBR) scale were used [27].

### 4.2. Preparation of Material for Research

During the surgery, a fragment of the tumor (test sample) and tissue surrounding the tumor (control sample) were collected from the patients qualified for the study. The samples were subjected to histopathological evaluation to confirm the presence or absence of neoplastic cells. The collected tissue fragments were placed in sterile containers in an RNA-later solution (Invitrogen, Carlsbad, CA, USA) and stored at −20 °C.

### 4.3. Tissue Homogenization

The collected tissues were homogenized using the Precellys 24 homogenizer (Bertin-Instruments, Montigny-le-Bretonneux, France) with the Cryolys cooling option, enabling work with thermosensitive molecules. Tissue disintegration was achieved using a disintegrating material in the form of stainless steel beads (TK Biotech, Warszawa, Poland) placed in homogenized biological material.

### 4.4. RNA Isolation and cDNA Reverse Transcription

The collected tissue fragments were homogenized using a Precellys 24 homogenizer (Bertin-Instruments, Montigny-le-Bretonneux, France) with the Cryolys cooling option. The isolation of genetic material was performed using the modified Chomczyński and Sacchi method [36]. The quality and quantity of the isolated RNA was determined using a NanoDrop ND-1000 spectrophotometer (Thermo Fisher Scientific, Waltham, MA, USA). The reverse transcription process was performed according to the manufacturer’s instructions using the High-Capacity cDNA Reverse Transcription Kit (Applied Biosystem, Foster City, CA, USA).

The expression levels of the tested genes were determined using a 384-well TaqMan™^®^ Human *ABC* Transporter Array (Applied Biosystems, Foster City, CA, USA) based on the manufacturer’s protocol. The endogenous control in the presented studies was *18S*-Hs99999901_s1. The level of gene expression using the real-time PCR method was determined according to the previously described procedure [37].

The following gene expression levels were included in the analysis: *ABCA2*-Hs00242232_m1, *ABCA3*-Hs00184543_m1, *ABCB1*-Hs00184491_m1, *ABCB4*-Hs00188776_m1, *ABCB9*-Hs00608640_m1, *ABCC1*-Hs00219905_m1, *ABCC2*-Hs00166123_m1, *ABCC3*-Hs00358656_m1, *ABCC4*-Hs00195260_m1, *ABCC5*-Hs00981089_m1, *ABCC6*-Hs00184566_m1, *ABCC10*-Hs00375716_m1, *ABCC11*-Hs01090768_m1, and *ABCG2*-Hs00184979_m1. The results were analyzed as logRQ values of gene expression.

### 4.5. Statistical Analysis of Data

Statistica v.13.3, DisPlayr and GraphPad v.5.01 were used in the statistical analysis and graphic design (*p* < 0.05 was assumed statistically significant). The U Mann–Whitney test and the Kruskall–Wallis H test with multiple comparisons were used to calculate the differences in the expression level between genes and the r-Spearman coefficient was used with a heatmap correlation matrix for correlation analysis.

The data contained in The Cancer Genome Atlas (TCGA) data were analyzed using Ualcan (http://ualcan.path.uab.edu/ (accessed on 15 November 2022)) [38,39]. The data contained in The Cancer Genome Atlas (TCGA) and SCAN-B data were analyzed using the Breast Cancer Gene Expression Miner v4.8 (bc-GenExMiner v4.8, http://bcgenex.ico.unicancer.fr/BC-GEM/GEM-Accueil.php?js=1 (accessed on 1 December 2022)) [40,41].

## Figures and Tables

**Table 1:** The expression levels of selected genes from the *ABC* family—descriptive statistics.

Genes	The Descriptive Statistics		
	Mean [logRQ]	Median [logRQ]	SD [logRQ]
*ABCA2*	−0.191886	−0.256099	0.953895
*ABCA3*	−0.093406	−0.097453	0.811485
*ABCB1*	−0.302247	−0.364516	1.046728
*ABCB4*	−0.381887	−0.510042	1.090308
*ABCB9*	−0.033501	0.022634	0.887895
*ABCC10*	−0.062088	0.010299	0.648125
*ABCC11*	−0.262637	−0.399027	1.632714
*ABCC1*	−0.182129	−0.162096	0.697759
*ABCC2*	−0.216629	−0.275724	1.057454
*ABCC3*	0.038216	0.056714	0.789761
*ABCC4*	−0.046965	−0.004365	0.961714
*ABCC5*	−0.210334	−0.154902	0.995382
*ABCC6*	−0.949418	−0.987163	1.085124
*ABCG2*	−0.628239	−0.661544	0.962047

**Table 2:** Levels of statistically significant differences in the expression of the examined genes in patients classified into the SBR1, SBR2, and SBR3 groups (H Kruskal–Wallis test with multiple comparison) and descriptive statistics. Statistically significant values are marked with *.

Gene	SBR1		SBR2		SBR3		*p* for Multiple Comparison
	Mean	SD	Mean	SD	Mean	SD	
*ABCA2*	−0.6129	1.00908	−0.0526	1.33483	−0.2077	0.77625	SBR1*SBR2 = 0.001720 * SBR1*SBR3 = 0.000002 * SBR2*SBR3 = 0.084
*ABCA3*	0.1042	0.82632	−0.0923	0.67787	−0.1137	0.83441	SBR1*SBR2 = 0.012065 * SBR1*SBR3 = 0.000262 * SBR2*SBR3 = 0.603
*ABCB1*	0.1909	1.19809	−0.4505	0.75874	−0.3108	1.08079	SBR1*SBR2 = 0.000000 * SBR1*SBR3 = 0.000000 * SBR2*SBR3 = 0.336
*ABCB4*	−0.1702	0.84631	−0.3070	0.77300	−0.4408	1.13838	SBR1*SBR2 = 0.238 SBR1*SBR3 = 0.000001 * SBR2*SBR3 = 0.000001 *
*ABCB9*	−0.1013	0.86123	0.0134	0.81472	−0.0438	0.90357	SBR1*SBR2 = 0.223 SBR1*SBR3 = 1.000000 SBR2*SBR3 = 0.250
*ABCC10*	−0.0889	0.76072	−0.1453	0.60914	−0.0458	0.63978	SBR1*SBR2 = 1.000 SBR1*SBR3 = 0.510 SBR2*SBR3 = 0.005770 *
*ABCC11*	−0.3424	1.54712	0.2333	1.93180	−0.4189	1.50223	SBR1*SBR2 = 0.020891 * SBR1*SBR3 = 1.000 SBR2*SBR3 = 0.000000 *
*ABCC1*	−0.1346	0.66676	−0.2032	0.59844	−0.1843	0.71459	SBR1*SBR2 = 0.894 SBR1*SBR3 = 0.799 SBR2*SBR3 = 1.000
*ABCC2*	−0.4179	0.72293	−0.1101	0.81112	−0.2358	1.12648	SBR1*SBR2 = 0.030500 * SBR1*SBR3 = 1.000 SBR2*SBR3 = 0.000043 *
*ABCC3*	−0.13340	1.034263	0.03236	0.719029	0.04699	0.781132	SBR1*SBR2 = 0.692 SBR1*SBR3 = 0.279 SBR2*SBR3 = 1.000
*ABCC4*	0.4054	0.92363	0.1272	0.86296	−0.1213	0.97339	SBR1*SBR2 = 0.008312 * SBR1*SBR3 = 0.000000 * SBR2*SBR3 = 0.000001 *
*ABCC5*	0.2376	1.05409	−0.3055	0.82283	−0.2250	1.01726	SBR1*SBR2 = 0.000000 * SBR1*SBR3 = 0.000000 * SBR2*SBR3 = 0.727
*ABCC6*	−1.0264	1.39572	−0.8833	0.97740	−0.9630	1.09047	SBR1*SBR2 = 0.251 SBR1*SBR3 = 1.000 SBR2*SBR3 = 0.104
*ABCG2*	−0.5105	0.96400	−0.6190	0.79172	−0.6441	0.99699	SBR1*SBR2 = 1.000 SBR1*SBR3 = 0.334 SBR2*SBR3 = 0.311

There is no information on this clinical parameter in the TCGA database.

**Table 3:** Descriptive statistics including the level of significance of the difference in the expression of the studied genes in three groups of patients distinguished on the basis of the size of the primary tumor (the H Kruskal–Wallis test with multiple comparison). Statistically significant values are marked with *.

Gene	T1		T2		T3		*p* for Multiple Comparison
	Mean	SD	Mean	SD	Mean	SD	
*ABCA2*	−0.249	0.6788	−0.104	1.0164	−0.454	0.7854	T1*T2 = 1.000 T1*T3 = 0.000106 * T2*T3 = 0.000000 *
*ABCA3*	−0.054	0.7497	−0.062	0.8370	−0.219	0.7322	T1*T2 = 1.000 T1*T3 = 0.015588 * T2*T3 = 0.004266 *
*ABCB1*	−0.181	0.8550	−0.345	0.9619	−0.250	1.3266	T1*T2 = 0.002718 * T1*T3 = 0.000388 * T2*T3 = 0.523
*ABCB4*	−0.455	0.8863	−0.382	0.9247	−0.411	1.4576	T1*T2 = 0.258 T1*T3 = 0.018024 * T2*T3 = 0.000000 *
*ABCB9*	−0.109	0.8276	0.011	0.871	−0.138	0.9376	T1*T2 = 0.072 T1*T3 = 1.000 T2*T3 = 0.000091 *
*ABCC10*	−0.151	0.6413	−0.020	0.619697	−0.164	0.6882	T1*T2 = 0.003034 * T1*T3 = 1.000 T2*T3 = 0.000002 *
*ABCC11*	0.629	1.6187	−0.316	1.6083	−0.631	1.5318	T1*T2 = 0.00000 * T1*T3 = 0.00000 * T2*T3 = 0.000615 *
*ABCC1*	−0.179	0.6255	−0.178	0.6775	−0.207	0.7568	T1*T2 = 1.000 T1*T3 = 0.655 T2*T3 = 0.444
*ABCC2*	−0.067	0.8799	−0.210	1.1216	−0.312	0.8977	T1*T2 = 0.006164 * T1*T3 = 0.002114 * T2*T3 = 0.885
*ABCC3*	0.209	0.7487	0.098	0.7149	−0.247	0.9262	T1*T2 = 0.054 T1*T3 = 0.000000 * T2*T3 = 0.000000 *
*ABCC4*	−0.100	0.8229	0.006	0.9055	−0.184	1.1454	T1*T2 = 0.240 T1*T3 = 0.800 T2*T3 = 0.000077 *
*ABCC5*	−0.054	1.0241	−0.237	0.9864	−0.228	0.9796	T1*T2 = 0.005531 * T1*T3 = 0.017318 * T2*T3 = 1.000
*ABCC6*	−0.616	0.9689	−0.979	1.0944	−1.036	1.0852	T1*T2 = 0.000000 * T1*T3 = 0.000000 * T2*T3 = 1.000
*ABCG2*	−0.893	0.7430	−0.597	0.9739	−0.620	0.9741	T1*T2 = 0.000006 * T1*T3 = 0.001194 * T2*T3 = 0.876

**Table 4:** Characteristics of 68 patients qualified for the study.

Characteristic	Patients with TNBC (n = 68)
Age	
≤50	16 (≈23.53%)
>50	52 (≈76.47%)
Gender:	
Male	0 (0%)
Female	68 (100%)
Lymphovascular invasion	
Yes	17 (25%)
No	51 (75%)
Invasion of the fat tissue	
Yes	12 (≈17.65%)
No	56 (≈82.35%)
Tumor size	
T1	8 (≈11.76%)
T2	45 (≈66.18%)
T3	15 (≈22.06%)
Lymph nodes	
N0	32 (≈47.06%)
N1	21 (≈30.88%)
N2	10 (≈14.71%)
N3	5 (≈7.35%)
SBR grade	
SBR I	4 (≈5.88%)
SBR II	15 (≈22.06%)
SBR III	49 (≈72.06%)

## Data Availability

The data that support the findings of this study are available from the corresponding author upon reasonable request.

## Supplementary Materials

The following supporting information can be downloaded at: https://www.mdpi.com/article/10.3390/ijms24021257/s1.
