# Supplementary material for: Analysis of Changes in the Expression of Selected Genes from the ABC Family in Patients with Triple-Negative Breast Cancer
Source: Int J Mol Sci. 2023 Jan 9;24(2):1257. doi: 10.3390/ijms24021257 (PMC9860794; doi:10.3390/ijms24021257)
Supplement: Supplementary file 1 [file ijms-24-01257-s001.zip › Table S1.pdf]

Gene	pN0		pN1		pN2		pN3		p for multiple comparison
	Mean	SD	Mean	SD	Mean	SD	Mean	SD	
ABCA2	-0.2929	0.75861	0.0118	1.16756	-0.0307	0.97817	-0.8259	0.60322	pN0*pN1 = 0.000005*<br>pN0*pN2 = 0.004930*<br>pN0*pN3 = 1.000<br>pN1*pN2 = 0.000000*<br>pN1*pN3 = 0.000000*<br>pN2*pN3 = 0.000000*
ABCA3	-0.0551	0.76100	-0.0916	0.73370	-0.1423	1.10679	-0.3677	0.64780	pN0*pN1 = 1.000<br>pN0*pN2 = 0.000251*<br>pN0*pN3 = 0.000001*<br>pN1*pN2 = 0.004868*<br>pN1*pN3 = 0.000017*<br>pN2*pN3 =0.256
ABCB1	-0.3016	0.96989	-0.2646	1.06238	-0.1401	1.28819	-0.7911	0.87265	pN0*pN1 = 1.000<br>pN0*pN2 = 1.000<br>pN0*pN3 = 0.000000*<br>pN1*pN2 = 1.000<br>pN1*pN3 = 0.000000*<br>pN2*pN3 = 0.000000*
ABCB4	-0.3588	0.80753	-0.4440	1.02572	-0.1684	1.76358	-0.9646	0.88671	pN0*pN1 = 0.005667*<br>pN0*pN2 = 0.000963*<br>pN0*pN3 = 0.000000*<br>pN1*pN2 = 1.000<br>pN1*pN3 = 0.000006*<br>pN2*pN3 = 0.001317*
ABCB9	0.0165	0.87285	0.0777	0.91056	-0.3468	0.80542	-0.4396	0.75689	pN0*pN1 = 1.000<br>pN0*pN2 = 0.000000*<br>pN0*pN3 = 0.000000*<br>pN1*pN2 = 0.000000*<br>pN1*pN3 = 0.000000*<br>pN2*pN3 = 1.000
ABCC10	-0.0217	0.62406	-0.1013	0.63770	-0.1016	0.72526	-0.2149	0.58081	pN0*pN1 = 0.039046*<br>pN0*pN2 = 0.738<br>pN0*pN3 = 0.000503*<br>pN1*pN2 = 1.000<br>pN1*pN3 = 0.123<br>pN2*pN3 = 0.094
ABCC11	-0.2326	1.68186	-0.3431	1.57369	0.2450	1.55241	-1.2427	1.06935	pN0*pN1 = 1.000<br>pN0*pN2 = 0.000004*<br>pN0*pN3 = 0.000000*<br>pN1*pN2 = 0.000000*<br>pN1*pN3 = 0.000000*<br>pN2*pN3 = 0.000000*
ABCC1	-0.1566	0.66150	-0.1016	0.71148	-0.3185	0.73581	-0.5409	0.59517	pN0*pN1 = 0.327<br>pN0*pN2 = 0.000077*<br>pN0*pN3 = 0.000000*<br>pN1*pN2 = 0.000000*<br>pN1*pN3 = 0.000000*<br>pN2*pN3 = 0.001062
ABCC2	-0.1683	1.09339	-0.2491	0.96335	0.0856	1.08100	-0.8883	0.80209	pN0*pN1 = 1.000<br>pN0*pN2 = 0.005331*<br>pN0*pN3 = 0.000000*<br>pN1*pN2 = 0.003051*<br>pN1*pN3 = 0.000000*<br>pN2*pN3 = 0.000000*
ABCC3	0.0616	0.77072	0.0439	0.70771	0.0953	0.97695	-0.3272	0.66940	pN0*pN1 = 1.000<br>pN0*pN2 = 0.395<br>pN0*pN3 = 0.000000*<br>pN1*pN2 = 0.200<br>pN1*pN3 = 0.000001*<br>pN2*pN3 = 0.000000*
ABCC4	0.0974	0.90232	0.0693	0.91088	-0.6419	1.04390	-0.5391	0.84510	pN0*pN1 = 1.000<br>pN0*pN2 = 0.000000*<br>pN0*pN3 = 0.000000*<br>pN1*pN2 = 0.000000*<br>pN1*pN3 = 0.000000*<br>pN2*pN3 = 1.000
ABCC5	-0.1972	0.95652	-0.2255	0.90709	0.0010	1.23445	-0.7679	0.82960	pN0*pN1 = 1.000<br>pN0*pN2 = 0.263

									pN0*pN3 = 0.000000*<br>pN1*pN2 = 0.227<br>pN1*pN3 = 0.000000*<br>pN2*pN3 = 0.000000*
ABCC6	-0.9200	1.09419	-1.0002	1.12087	-0.6966	0.91345	-1.5966	0.91776	pN0*pN1 = 0.248<br>pN0*pN2 = 0.000054*<br>pN0*pN3 = 0.000000*<br>pN1*pN2 = 0.000000*<br>pN1*pN3 = 0.000000*<br>pN2*pN3 = 0.000000*
ABCG2	-0.6243	0.97418	-0.6452	0.81671	-0.4338	1.05417	-1.0109	1.07217	pN0*pN1 = 1.000<br>pN0*pN2 = 0.037611*<br>pN0*pN3 = 0.000004*<br>pN1*pN2 = 0.179<br>pN1*pN3 = 0.000002*<br>pN2*pN3 = 0.000000*
