# Supplementary material for: Analysis of Changes in the Expression of Selected Genes from the ABC Family in Patients with Triple-Negative Breast Cancer
Source: Int J Mol Sci. 2023 Jan 9;24(2):1257. doi: 10.3390/ijms24021257 (PMC9860794; doi:10.3390/ijms24021257)
Supplement: Supplementary file 1 [file ijms-24-01257-s001.zip › Table S2.pdf]

Table S2. The level of significance of the difference in the expression of the studied genes in patients classified into pN0, pN1, pN2, pN3 groups by the metastases to the regional lymph nodes obtained from the Ualcan database

**Gene**	**p for multiple comparison**
ABCA2	pN0*pN1= 2.056300E-02*<br>pN0*pN2= 1.076240E-01<br>pN0*pN3= 7.832600E-01<br>pN1*pN2= 9.599800E-01<br>pN1*pN3= 1.975800E-01<br>pN2*pN3= 2.838000E-01
ABCA3	pN0*pN1= 3.642600E-01<br>pN0*pN2= 7.143800E-01<br>pN0*pN3= 4.725000E-01<br>pN1*pN2= 3.237800E-01<br>pN1*pN3= 2.324200E-01<br>pN2*pN3= 7.173600E-01
ABCB1	pN0*pN1= 1.865030E-01<br>pN0*pN2= 2.541200E-01<br>pN0*pN3= 2.955800E-02*<br>pN1*pN2= 9.331600E-01<br>pN1*pN3= 1.633380E-01<br>pN2*pN3= 2.128000E-01
ABCB4	pN0*pN1= 9.786400E-01<br>pN0*pN2= 2.532200E-01<br>pN0*pN3= 4.893600E-01<br>pN1*pN2= 2.181200E-01<br>pN1*pN3= 4.667600E-01<br>pN2*pN3= 1.314810E-01
ABCB9	pN0*pN1= 7.196100E-02<br>pN0*pN2= 2.118800E-03*<br>pN0*pN3= 9.435600E-03*<br>pN1*pN2= 4.883900E-02*<br>pN1*pN3= 1.794000E-01<br>pN2*pN3= 6.776200E-01
ABCC1	pN0*pN1= 5.647400E-01<br>pN0*pN2= 5.022200E-01<br>pN0*pN3= 9.831600E-01<br>pN1*pN2= 2.942000E-01<br>pN1*pN3= 7.104200E-01<br>pN2*pN3= 6.317800E-01

ABCC2	pN0*pN1= 1.595620E-01<br>pN0*pN2= 8.430600E-01<br>pN0*pN3= 4.282400E-01<br>pN1*pN2= 3.422600E-01<br>pN1*pN3= 8.944000E-01<br>pN2*pN3= 4.711000E-01
ABCC3	pN0*pN1= 7.804400E-01<br>pN0*pN2= 9.330800E-01<br>pN0*pN3= 4.933800E-02*<br>pN1*pN2= 9.161800E-01<br>pN1*pN3= 8.744100E-02<br>pN2*pN3= 1.169820E-01
ABCC4	pN0*pN1= 3.990800E-01<br>pN0*pN2= 1.984000E-01<br>pN0*pN3= 6.776600E-01<br>pN1*pN2= 4.150000E-01<br>pN1*pN3= 8.853400E-01<br>pN2*pN3= 4.045600E-01
ABCC5	pN0*pN1= 8.204700E-03*<br>pN0*pN2= 5.581200E-01<br>pN0*pN3= 6.538400E-01<br>pN1*pN2= 7.254000E-02<br>pN1*pN3= 3.888400E-02*<br>pN2*pN3= 8.820800E-01
ABCC6	pN0*pN1= 2.381600E-01<br>pN0*pN2= 9.054200E-01<br>pN0*pN3= 2.699600E-03*<br>pN1*pN2= 2.708400E-01<br>pN1*pN3= 6.283200E-03*<br>pN2*pN3= 5.382200E-02
ABCC10	pN0*pN1= 2.147500E-02*<br>pN0*pN2= 1.443250E-03*<br>pN0*pN3= 6.508400E-02<br>pN1*pN2= 2.386200E-02*<br>pN1*pN3= 4.444400E-01<br>pN2*pN3= 1.950810E-01
ABCC11	pN0*pN1= 1.100810E-01<br>pN0*pN2= 4.653600E-01<br>pN0*pN3= 1.450310E-02*<br>pN1*pN2= 1.108220E-01<br>pN1*pN3= 2.216200E-03*<br>pN2*pN3= 1.453790E-01
ABCG2	pN0*pN1= 7.565600E-01

	pN0*pN2= 6.357200E-01<br>pN0*pN3= 6.976800E-01<br>pN1*pN2= 4.294400E-01<br>pN1*pN3= 8.898600E-01<br>pN2*pN3= 4.077200E-01
