# Supplementary figures and images for: Analysis of Changes in the Expression of Selected Genes from the ABC Family in Patients with Triple-Negative Breast Cancer
Source: Int J Mol Sci. 2023 Jan 9;24(2):1257. doi: 10.3390/ijms24021257 (PMC9860794; doi:10.3390/ijms24021257)

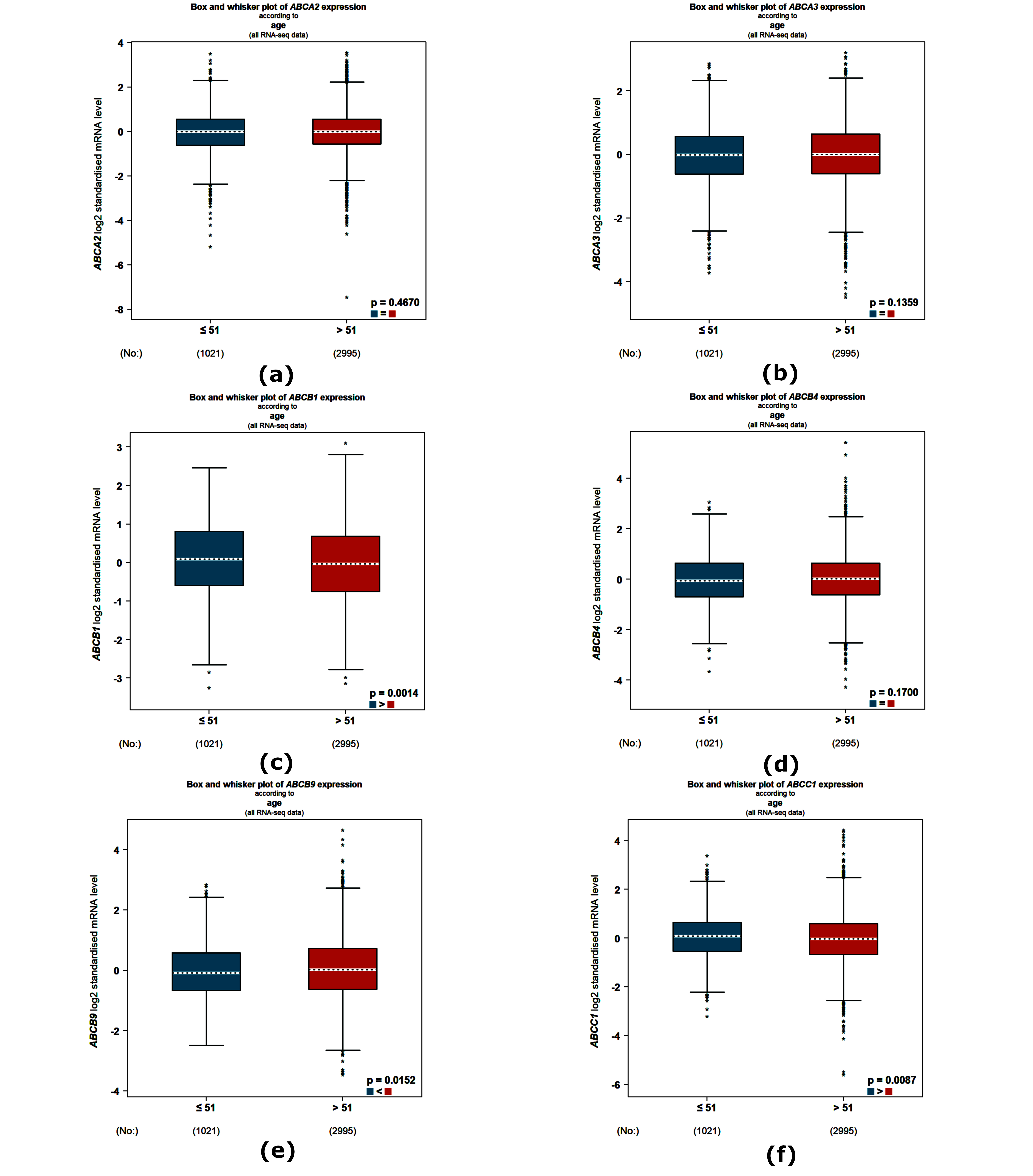

Supplement: Supplementary file 1 [file ijms-24-01257-s001.zip › Figure S1.jpg]

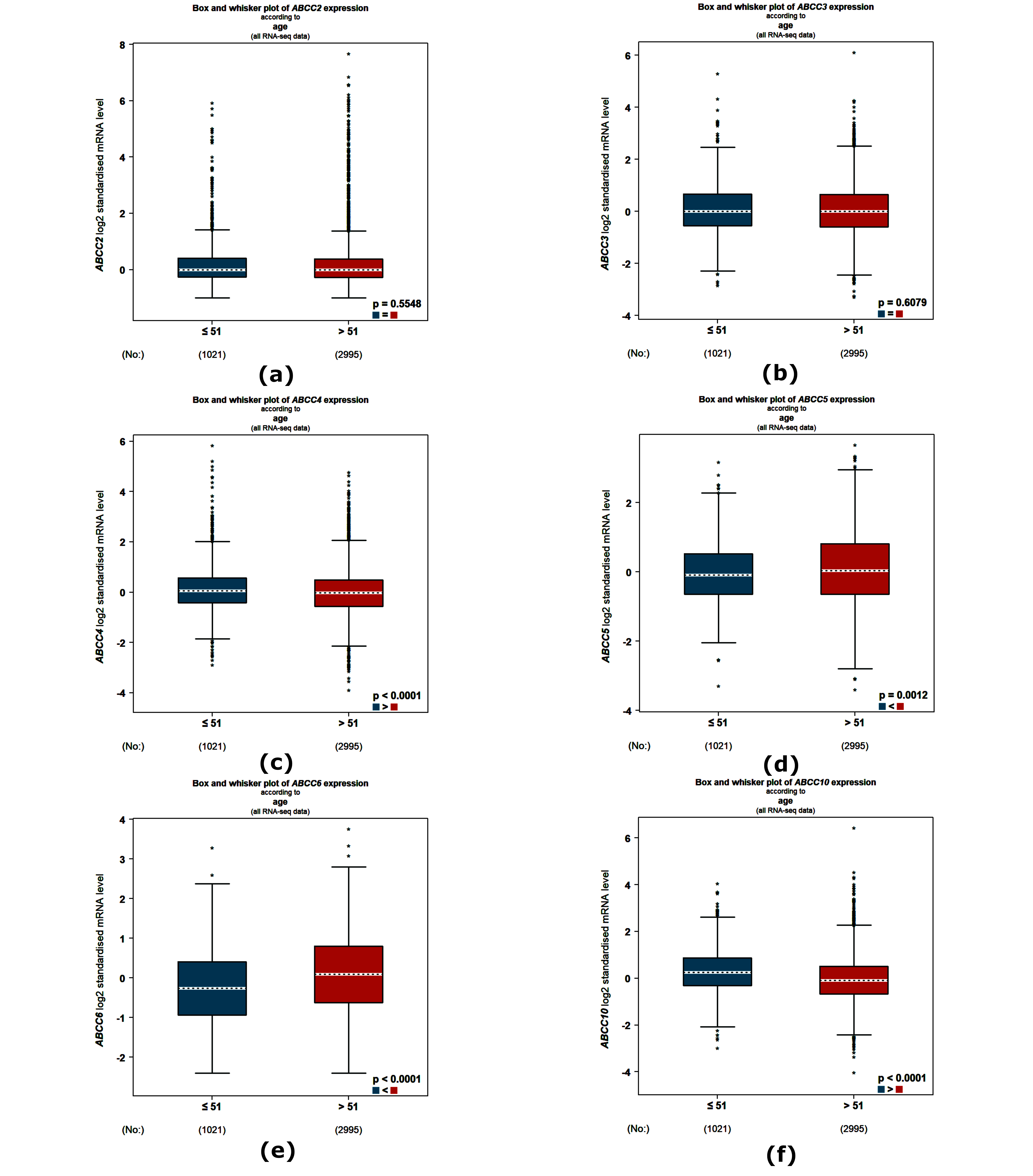

Supplement: Supplementary file 1 [file ijms-24-01257-s001.zip › Figure S2.jpg]

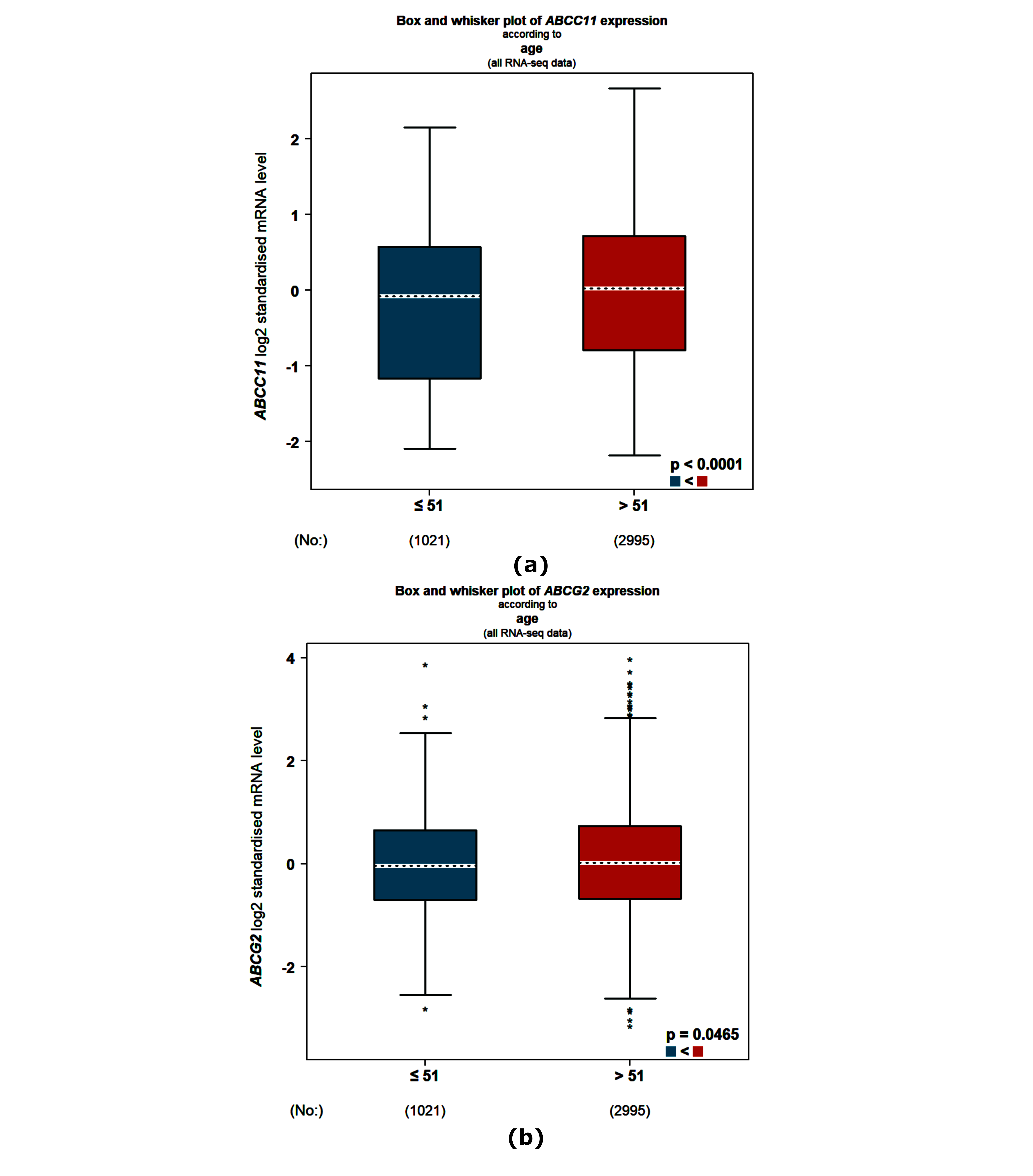

Supplement: Supplementary file 1 [file ijms-24-01257-s001.zip › Figure S3.jpg]

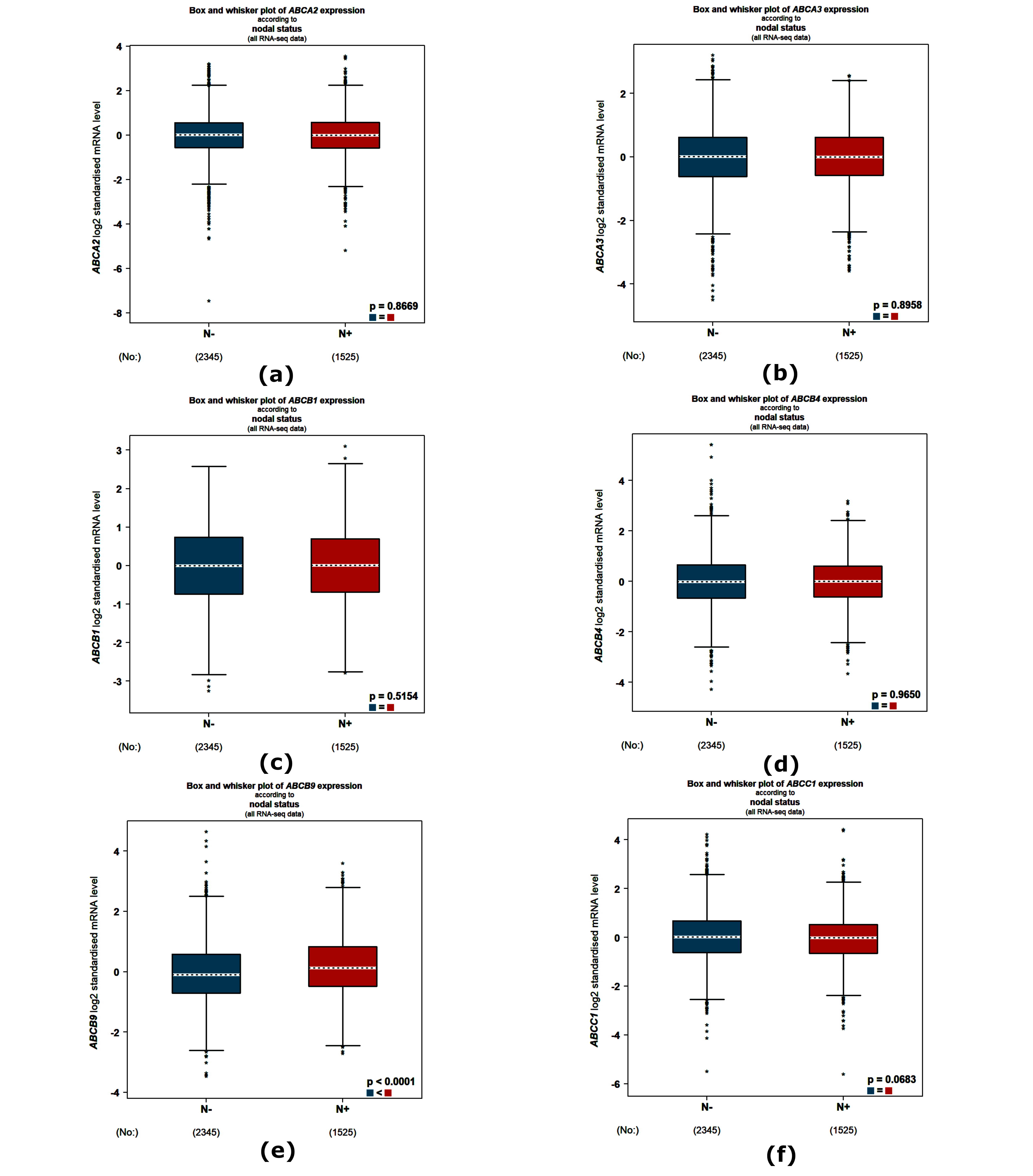

Supplement: Supplementary file 1 [file ijms-24-01257-s001.zip › Figure S4.jpg]

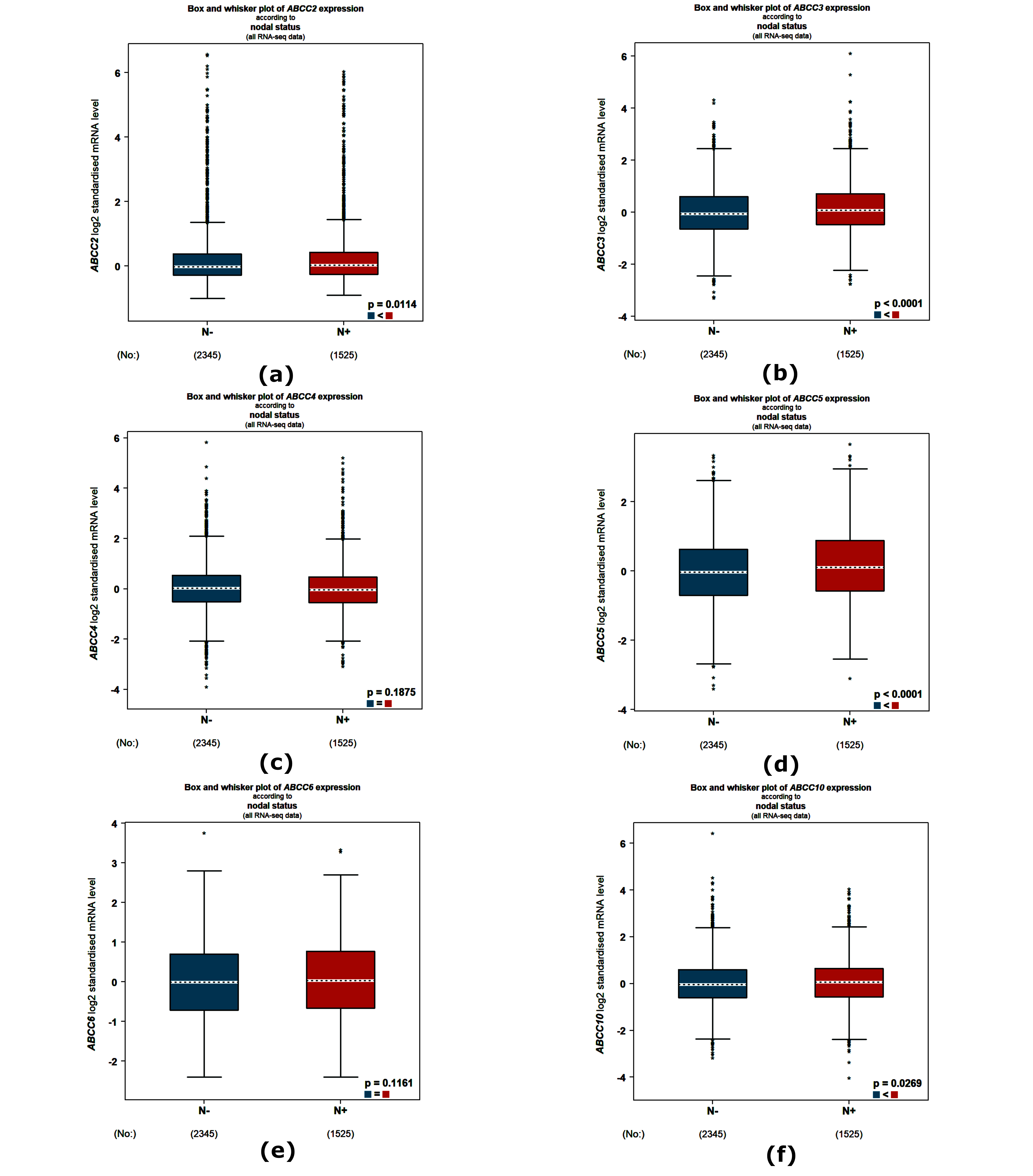

Supplement: Supplementary file 1 [file ijms-24-01257-s001.zip › Figure S5.jpg]

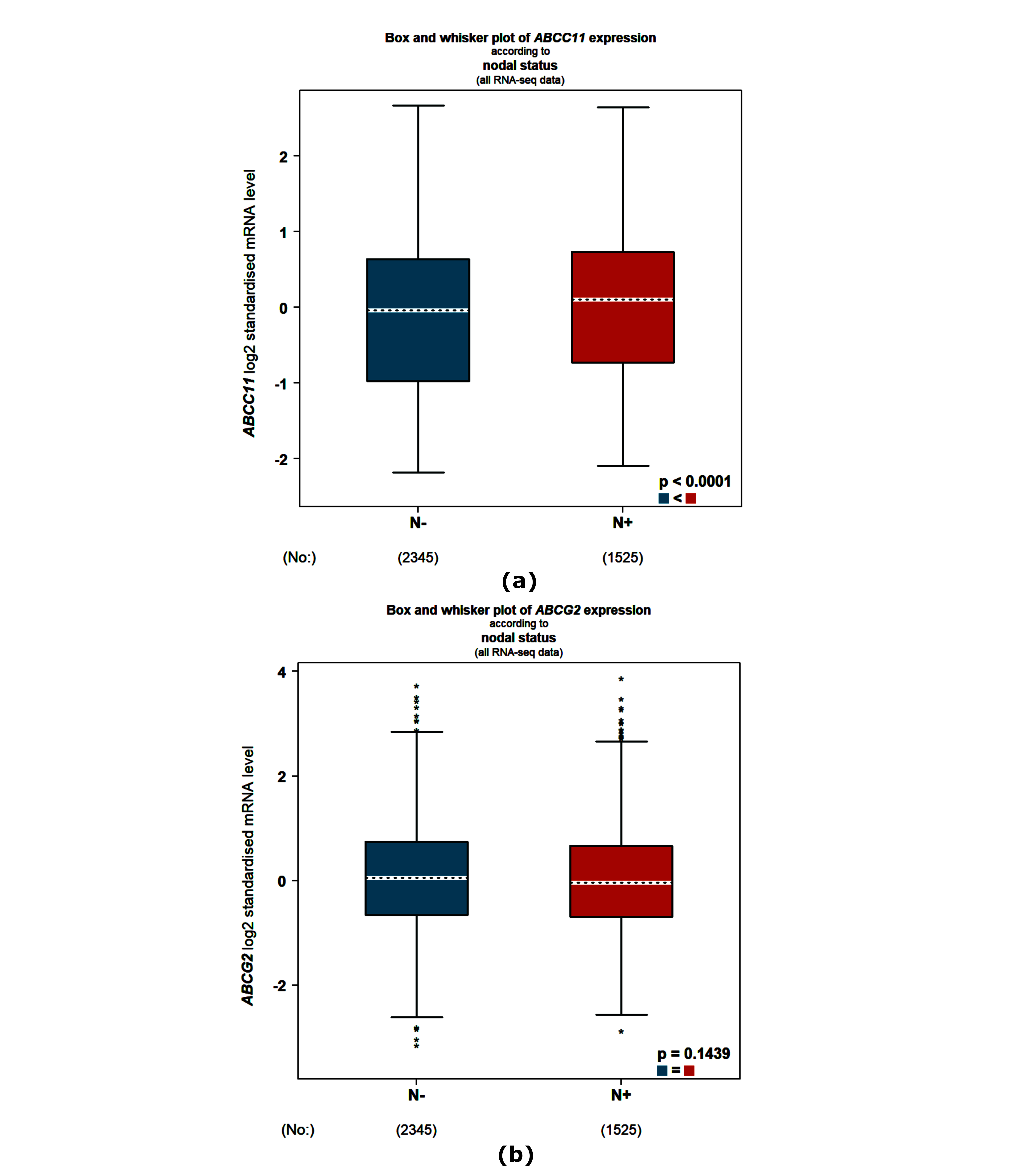

Supplement: Supplementary file 1 [file ijms-24-01257-s001.zip › Figure S6.jpg]
